# Does Radioiodine Therapy in Patients with Differentiated Thyroid Cancer Increase the Frequency of Another Malignant Neoplasm?

**DOI:** 10.5402/2011/708343

**Published:** 2011-08-14

**Authors:** Renata Midori Hirosawa, Monica Marivo, Juliana de Moura Leite Luengo, Jose Vicente Tagliarini, Emanuel Cellice Castilho, Mariangela de Alencar Marques, Yoshio Kiy, Marilia Martins Silveira Marone, Liciana Vaz de Arruda Silveira, Glaucia Maria Ferreira da Silva Mazeto

**Affiliations:** ^1^Departamento de Clinica Medica, Faculdade de Medicina (FMB), UNESP, Rubiao Junior s/n, Botucatu, 18618-000 São Paulo, Brazil; ^2^Departamento de Oftalmologia e Otorrinolaringologia, Faculdade de Medicina (FMB), UNESP, Rubiao Junior s/n, Botucatu, 18618-000 São Paulo, Brazil; ^3^Departamento de Patologia, Faculdade de Medicina (FMB), UNESP, Rubiao Junior s/n, Botucatu, 18618-000 São Paulo, Brazil; ^4^Servico de Medicina Nuclear, Faculdade de Medicina (FMB), UNESP, Rubiao Junior s/n, Botucatu, 18618-000 São Paulo, Brazil; ^5^Servico de Medicina Nuclear, Santa Casa de Sao Paulo, Rua Cesario Mota Junior, 112 ,Vila Buarque, 01221-020 São Paulo, Brazil; ^6^Departamento de Bioestatistica, Instituto de Biociencias, UNESP, Rubiao Junior s/n, Botucatu, 18618-000 São Paulo, Brazil

## Abstract

*Objectives*. To compare the frequency of another primary malignancy in patients with differentiated thyroid carcinoma (DTC) who received radioiodine therapy or not (^131^I). *Material and Methods*. 168 cases of DTC patients were retrospectively evaluated as to the frequency of another neoplasia by comparing patients with and without it, taking into account clinical, laboratory, and therapeutic parameters. *Results*. Another primary malignancy occurred in 8.9% of patients. Of these, 53.3% showed the malignancy before ^131^I and 46.7% after it. By comparing both groups, the age at the moment of diagnosis of another neoplasia was 46.1 ± 20.2 years for the group before ^131^I therapy and of 69.4 ± 11.4 years for the group after it (*P* = 0.02). Of the 148 patients treated with ^131^I, 4.7% developed another malignancy. The latter were older (61 ± 17 years) than those who did not show another cancer type (44.1 ± 14.2 years) (*P* < 0.05). *Conclusion*. The frequency of another neoplasia found after ^131^I was similar to that found before ^131^I.

## 1. Introduction

Differentiated thyroid carcinomas (DTC) are the most frequent malignant endocrine neoplasias [1, 2], and when properly treated, they show good prognosis and similar life expectancy to that of the general population [3].

Total thyroidectomy followed by radioiodine ablation (^131^I), associated with TSH suppression therapy with levothyroxine, is the initial treatment recommended for the majority of patients [4, 5].

Thyroid ablation by ^131^I fosters the followup of patients with DTC since it increases serum thyroglobulin specificity as a tumor marker. It also fosters early metastasis detection by means of whole-body scanning (WBS) after an uptake [6]. 

Recent studies have reported increased risk for developing a second cancer after radioiodine therapy [7–9]. On the other hand, some authors have suggested that patients with DTC per se show increased incidence of neoplasias that are unrelated to ^131^I therapy, thus suggesting another common etiology and/or genetic mechanism rather than a causal relationship [10, 11].

Hence, the present study aimed at evaluating the frequency of another primary malignancy, in addition to DTC, and its temporal relation to ^131^I, in patients assisted at a specialized outpatient unit of a university hospital. 

## 2. Material and Methods

The medical records of 168 patients with DTC who initiated followup at the Thyroid Neoplasia Outpatient Unit of the Botucatu School of Medicine, UNESP from 1970 to 2008 were retrospectively evaluated. 

The following data were obtained: gender, age, age at the time of thyroidectomy, DTC histological type, follow-up period, radioiodine therapy performance, accumulated dose of ^131^I, presence of a second malignancy, age at its diagnosis, temporal relationship between the diagnosis of the second malignancy and ^131^I dose, and the second type of malignancy presented. 

Of the 168 patients, eight were not submitted to ^131^I, and 12 did not have information about the total ^131^I dose. Thus, the number of patients effectively treated with radioiodine was 148. They were classified into two groups (with and without another neoplasia after ^131^I use) and then compared in relation to gender, age at thyroidectomy, histological type of DTC, follow-up period, accumulated dose of ^131^I, age at the diagnosis of another malignancy, and the second type of malignancy presented.

The patients who developed another neoplasia were also classified into two groups: the first group showed a second malignancy before the ^131^I dose and the second group showed it after the ^131^I dose. Such two groups were also compared with respect to the abovementioned parameters. 

### 2.1. Statistical Analysis

Excel sheets (Microsoft Corporation, EUA) and the Statistical Analysis System (SAS) software package, version 9.2, were used for statistical analysis. Results were expressed in terms of frequencies, mean ± standard deviation, or median (minimum and maximum values). Clinical, laboratory, and development data were compared by using the most appropriate statistical tests to each case. Student's *t*-test for independent samples, the Chi-square test, and Fisher's exact test were utilized. Logistic regression, from which the odds ratio was obtained, and generalized linear models, with gamma error and a log-link function, were also fitted. 95% confidence intervals and a 5% level of significance (*P* < 0.05) were used. 

## 3. Results

The patients' general, clinical, epidemiological, and therapeutic characteristics, are shown in Table 1. Fifteen patients (8.9%) showed another neoplasia in addition to DTC. Of these, eight (53.3%) did not receive ^131^I or showed the second malignancy before ^131^I use, and seven (46.6%) showed it after ^131^I therapy. By comparing these two groups, age at the diagnosis of the second neoplasia was smaller for patients who developed it before ^131^I than in the group in which another malignancy was observed after radioiodine therapy (*P* = 0.02). No significant differences were observed with respect to gender, age at thyroidectomy, follow-up period, or histological type of DTC between the two groups (Table 2).

The neoplasias diagnosed before and after ^131^I therapy are specified in Table 3.

Of the 148 patients who received a known accumulated dose of ^131^I, seven (4.7%) developed a second malignancy after treatment. The median time between ^131^I therapy and diagnosis of second malignancy was 60 months, with minimum of 6 months and maximum of 275 months. Age at thyroidectomy was greater in the group of individuals who developed another malignancy than in those who did not show it (*P* = 0.006). No statistically significant differences were observed with respect to gender, follow-up period, accumulated ^131^I dose, or histological type of DTC between the two groups (Table 4). 

## 4. Discussion

The association of radioiodine therapy with the development of a second neoplasia in patients with DTC has been controversial. In our service, the frequency of other primary neoplasias was 8.9%, which is an apparently high percentage when taking into account that the estimated incidence of malignant neoplasias for the general population in São Paulo state for the years 2010 and 2011 is approximately 313 per year for every 100,000 inhabitants [12]. 

Increased risk for a second neoplasia in patients with DTC has been reported. Sandeep et al. analyzed over 39,000 patients with thyroid carcinoma and observed 30% risk increase for developing a second malignancy as compared to the general population. Additionally, greater occurrence of thyroid cancer in patients with other types of neoplasias has been found [8]. 

In the present study, it was observed that, of the patients showing another neoplasia, similar percentages had (46.6%) or had not (53.3%) received a therapeutic dose of ^131^I before the other neoplasm. These findings are in agreement with those reported by Bhattacharyya et al., who observed that the use of radioiodine did not increase risk for developing a second neoplasia by evaluating over 29,000 patients with DTC [11]. Similarly, Verkooijen et al. observed that the increased incidence of a second neoplasia was not related to ^131^I therapy, thus suggesting the nonexistence of a causal relationship between the two events [10]. 

Other studies, however, have shown increased risk for a second malignancy related to radioiodine therapy. In a prospective study on 6,841 European patients with DTC, Rubino et al. reported 27% risk for developing a second neoplasia, which is a significant increase in relation to the general population in the studied countries [7]. Those authors also observed increased risk for both solid tumors and leukemia, according to the accumulated dose of radioiodine administered. In 2008, Brown et al. published a prospective study that followed over 30,000 American patients with DTC for approximately 103 months and also reported significantly increased risk for a second neoplasia. Such risk changed according to age at diagnosis and radioactive iodine use [9]. A recent meta-analysis published in 2009 studied over 16,000 American and European patients and found increased risk for developing a second neoplasia in patients receiving ^131^I therapy. Such risk was particularly higher for the development of leukemia and linearly associated with the accumulated radioiodine dose. However, previous exposure to ^131^I therapy was not related to increased risk for neoplasias in the breast, central nervous system, colon, rectum, kidneys, or stomach of patients with DTC as compared to the general population [13]. 

The differences in the findings from different studies may be related to a number of factors, among which are the particular characteristics of the studied populations. Some factors, such as genetic profile and environmental characteristics, certainly influence the prevalence of different types of cancer. The role played by genetic heredity has been particularly studied. Several loci have recently been associated with some types of cancer and reported to increase their risk [14]. A study conducted on 9.6 million people found a greater association with hereditary factors in cancers in the thyroid (53%), endocrine system (25%), testis (23%), breasts (20%), and melanoma (20%). However, other types of cancers, such as those in the nervous system, colon, rectum, non-Hodgkin lymphoma, and lungs, showed only slight agreement [15]. In general, the specific role played by each gene locus in malignancy induction seems to be relatively small [14]. The phenomenon of incomplete alleles of cancer susceptibility and environmental exposure could be responsible for these findings. Hence, depending on the environment, an individual with high genetic risk for developing a neoplasia may never show it, while another individual at low risk could eventually develop it [16, 17]. Among environmental factors, the possible influence of disruptors is noteworthy. Exposure to such elements seems to play an important role in the occurrence of certain cancer types [18]. The group of molecules identified as disruptors is heterogeneous and includes synthetic products such as industrial solvents/lubricants and their byproducts [polychlorinated biphenyls (PCBs), polybrominated biphenyls (PBBs), dioxins], plastics [bisphenol A (BPA)], plasticizers (phthalates), pesticides [methoxychlor, chlorpyrifos, dichlorodiphenyltrichloroethane (DDT)], fungicides (vinclozolin), and pharmaceutical agents [diethylstilbestrol (DES)]. Natural compounds present in humans as well as animal foods (e.g., phytoestrogens, including genistein and coumestrol) can also work as disruptors [19, 20]. Depending on age and exposure time, studies show that some disruptors can change mechanisms that regulate cell proliferation and tissue organization patterns, and such alterations may also be associated with the development of neoplastic lesions. Disruptors, such as DDT, BPA, DES, seem to be associated with breast and prostate cancer [21–26].

In addition to the aforementioned factors, which affect the general population, we could question how much nutritional habits and iodine sufficiency influence the occurrence of other neoplasias, specifically in patients with DTD treated by ^131^I. The uptake of iodine by the thyroid tissue is mediated by sodium-iodide symporter (NIS), cloned in 1996 [27]. Although it is classic in the thyroid, NIS is also found in several other tissues that capture iodine, such as the stomach, salivary glands, lactating breasts, thymus, nasal mucosa, lachrymal glands, and placenta [28–30]. While the NIS-mediated uptake of radioiodine by normal or neoplastic thyroid cells depends, among other factors, on the concentration of organified intracellular iodine [31], the interferents affecting the uptake of that element in other organs are not yet known. Since the induction of malignancy by radioactive iodine must occur through exposure of tissues to that radiopharmaceutical, the effect of iodine sufficiency or deficiency on the uptake of ^131^I in nonthyroid tissues is questionable. Hence, we may be able to hypothesize that different populations with diverse genetic and environmental characteristics and different iodine sufficiency could respond differently to radioactive iodine with respect to the induction of another neoplasia. 

In this study, the studied characteristics (gender, follow-up period, histological type, age at diagnosis of another neoplasia) showed no statistical differences when comparing the group that developed a second malignancy before ^131^I use and the group that developed another neoplasia after ^131^I therapy. The only parameter showing significance was age at diagnosis of another neoplasia. 

When comparing the patients who underwent radioiodine therapy but did not develop a second neoplasia (141) to those who were also submitted to ^131^I and later developed another malignancy (7), again no differences were found for the following studied parameters: gender, follow-up period, histological type, and accumulated ^131^I dose. Additionally, the median accumulated ^131^I dose in patients who did not develop a second neoplasia (200 mCi) was even higher in relation to those showing another malignancy (175 mCi). The only parameter that showed statistically significant differences was age at thyroidectomy, thus suggesting that older patients at the time of surgery could be at increased risk for developing a second malignancy. The causes for this finding remain to be clarified. With respect to other primary malignancies found, no predominance of any specific neoplasia was observed. 

Our study showed limitations, such as its retrospective character and a short follow-up period (approximately 6.5 years). Moreover, we did not do an active screening of other cancers, considering the retrospective nature of the study, such a procedure impractical to monitor all patients with differentiated thyroid cancer, which would burden our health system. Nevertheless, our findings, as previously described in other studies, can suggest that, at least in certain populations, the occurrence of another malignancy after ^131^I does not have a causal relationship with such treatment. Hence, patients with DTC could have increased incidence of a second neoplasia which is not related to ^131^I therapy, thus more probably suggesting a common etiology and/or a genetic mechanism rather than a causal relationship between the two tumors. 

## Figures and Tables

**Table 1:** Clinical, epidemiological, and therapeutic characteristics of 168 patients with differentiated thyroid carcinoma (DTC).

Clinical, epidemiological, and therapeutic characteristics	
Females *n* (%)	Female: 142 (84.7)
Age* (years)	45.15 ± 15.32
Follow-up period** (months)	77.5 (1; 468)
Papilliferous carcinoma *n* (%)	137 (81.5)
Radioiodine therapy *n* (%)	160 (95.2)
Radioiodine dose** (mCi)	200 (30; 870)
Another neoplasia *n* (%)	15 (8.93)

*mean ± standard deviation; **median (minimum value; maximum value).

**Table 2:** Clinical and epidemiological characteristics of 15 patients with differentiated thyroid carcinoma and another malignancy.

Clinical and epidemiological characteristic	Another malignancy		*P*
	Postiodine *N* = 7 (46.66%)	Preiodine *N* = 8 (53.33%)	
Males *N* (%)	4 (57.14)	3 (37.5)	0.6193
Age at thyroidectomy (years)*	61 ± 17	51 ± 13.7	0.2295
Follow-up period (months)**	75 (48; 336)	56.5 (20; 60)	0.1505
Age at diagnosis of a second neoplasia* (years)	69.4 ± 11.4	46.1 ± 20.2	**0.0187**
Classic nonpapilliferous histological type *N* (%)	3 (42.85)	2 (25)	0.6084

*mean ± standard deviation; **median (minimum value; maximum value).

**Table 3:** Types of other neoplasias diagnosed in 15 patients with differentiated thyroid carcinoma.

PRE ^131^I	POST ^131^I
2 breast adenocarcinomas	2 skin spinocellular carcinomas
1 chronic myeloid leukemia	1 prostate adenocarcinoma
1 lymphoma	1 colon adenocarcinoma
1 rectal adenocarcinoma	1 endometrial adenocarcinoma
1 Palatal spinocellular carcinoma	1 rectal carcinoma
1 testicular seminoma	1 lung carcinoma
1 endometrial adenocarcinoma	

**Table 4:** Clinical, epidemiological, and therapeutic characteristics of 148 patients with differentiated thyroid carcinoma who received a therapeutic dose of radioactive iodine and showed another postiodine malignancy or not.

Clinical and epidemiological data	Another postradioactive iodine malignancy		Odds ratio	IC 95%	*P*
	Yes *N* = 7 (4.7%)	No *N* = 141 (95.3%)			
Males *N* (%)	4 (57.1)	21 (14.9)	0.123	0.015–1.026	0.0528
Age at thyroidectomy (years)*	61 ± 17	44.08 ± 14.2	**1.133**	**1.036**–**1.239**	**0.0061**
Follow-up period (months)**	75 (48; 336)	74 (9; 468)	1.013	1.000–1.026	0.0514
Accumulated dose of ^131^I (mCi)**	175 (100; 300)	200 (30; 870)	0.994	0.985–1.002	0.1375
Classic nonpapilliferous histological type *N* (%)	3 (42.8)	59 (41.8)	2.350	0.311–17.742	0.4073

*mean ± standard deviation; **median (minimum value; maximum value).
